# Optical kicking of liquid droplets for sample delivery in ultrafast soft X-ray experiments

**DOI:** 10.1107/S1600577525005430

**Published:** 2025-07-21

**Authors:** Zeinab Ebrahimpour, Dan Cojoc, Emiliano Principi, Riccardo Mincigrucci

**Affiliations:** a FERMI Elettra-Sincrotrone Trieste Area Science Park Basovizza 34149Trieste Italy; b CNR – Istituto Officina Dei Materiali Area Science Park Basovizza 34149Trieste Italy; University College London, United Kingdom

**Keywords:** ultra-fast soft X-ray experiments, liquid sample delivery, water droplets, optical impulse force, Hertz–Knudsen equations

## Abstract

We present a technique based on the optical force of a femtosecond laser acting on liquid micro-droplets for their precise manipulation in a vacuum, enabling an efficient sample delivery system for soft X-ray experiments. Conventional liquid jet methods, which are utilized in soft X-ray experiments, consume large sample volumes and offer limited control over droplet trajectories, leading to significant sample waste. Our approach uses optical forces from a femtosecond-pulsed focused laser to deflect free-falling droplets, guiding them with high precision toward the interaction region. This significantly reduces sample waste while enabling real-time control over droplet positioning. To understand the behavior of droplets in vacuum and their interaction with the focused laser beam, we employ theoretical analysis and numerical simulations. Hertz–Knudsen equations describe the thermodynamics of free-falling and deflected droplets, allowing estimation of their temperature and size as a function of time and position. The optical force acting on the droplets is determined using the transfer matrix method and Lorenz–Mie theory. The proposed technique provides fine tuning over delivery time and thermodynamic properties of the liquid sample, offering a promising platform for investigating supercooled liquid micro-droplets and phase transitions. It is a particularly well suited liquid sample delivery method for ultrafast X-ray experiments using tabletop sources, as well as current and future free-electron laser and high harmonic generation facilities.

## Abstract 2

Optical force-based control of liquid micro-droplets in vacuum enables precise, low-waste sample delivery for ultrafast soft X-ray experiments.

## 1. Introduction

Micrometre-sized liquid droplets in a vacuum system offer a promising platform for studying supercooled liquids and phase transition processes at low temperatures, reaching as low as *T* = 200 K for water (Sosso *et al.*, 2016 ▸; Kim *et al.*, 2017 ▸). However, experiments under these conditions face significant challenges due to the small sample size, the suppression of heterogeneous nucleation sites, and rapid evaporative cooling (*e.g.* ∼10^6^ K s^−1^ for water droplets below 10 µm), which complicates experimental setup and control. To conduct experiments on liquid samples in vacuum conditions, a reliable sample delivery system is essential. Recently, flat liquid jet sample delivery systems have been proposed for soft X-ray experiments, enabling the study of liquid samples, such as aqueous metal solutions, using compact tabletop sources (Gallo *et al.*, 2024 ▸; Smith *et al.*, 2020 ▸; Gnewkow *et al.*, 2024 ▸; Holburg *et al.*, 2025 ▸). Liquid jets consume large volumes of samples, which can be problematic when working with expensive samples or rare biological specimens. Additionally, they often provide a limited control over the trajectory of the droplets, making it difficult to ensure precise synchronization with pump–probe sources used in ultrafast soft X-ray spectroscopic studies.

In this work, we propose an optical force based method for manipulating liquid droplets in vacuum, introducing a conceptual design for efficient and adaptive sample delivery. Supported by simulations, this approach provides precise guidance of droplets from generation to the interaction point. The system uses femtosecond laser pulses to induce optical forces that precisely deflect free-falling droplets. The droplets’ thermodynamic evolution is quantified using Hertz–Knudsen equations, which model temperature and dimensional changes over time. Meanwhile, laser-droplet interactions are characterized through transfer matrix methodology. This integrated approach ensures precise control over both droplet trajectories and thermodynamic parameters, significantly enhancing experimental efficiency in vacuum environments.

Optical kicking presents multiple significant benefits for sample delivery. It conserves precious material by minimizing waste—particularly valuable when working with limited biological specimens. By precisely guiding droplets to ideal X-ray interaction positions, it substantially enhances the signal-to-noise ratio. The technique also offers remarkable versatility in delivery methods, allowing for dynamic sample positioning while simultaneously reducing common experimental challenges like contamination and clogging. These combined advantages establish optical kicking as a superior approach for soft X-ray sample delivery applications.

In the following, Section 1.1 introduces the principles of optical force, while Section 1.2 discusses droplet thermodynamical behavior in a vacuum.

### 1.1. Optical forces and particle manipulation

Arthur Ashkin’s pioneering work in the 1970s established the application of laser-induced optical forces, historically known as radiation pressure, for manipulating small particles (Ashkin, 1970 ▸; Ashkin & Dziedzic, 1971 ▸; Ashkin *et al.*, 1986 ▸; Ashkin, 1997 ▸). His experiments first demonstrated the control of silica particle dynamics with a focused beam, followed by the trapping of 20 µm glass spheres in air and at low pressures (Ashkin & Dziedzic, 1971 ▸). He also conducted liquid trapping experiments with silicon oil drops and demonstrated the trapping and levitation of micrometre-sized liquid droplets (Ashkin & Dziedzic, 1975 ▸). The manipulation of oil droplets at pressures as low as ∼3 × 10^−4^ Pa demonstrated that reduced dissipation in high-vacuum particle manipulation significantly enhances experimental sensitivity (Ashkin & Dziedzic, 1971 ▸).

Optical forces, typically in the picoNewton (pN) range, arise from momentum transfer when light interacts with a particle through absorption, reflection, refraction or scattering (Hu *et al.*, 2023 ▸). These forces enable the manipulation of particles by accelerating, decelerating, deflecting, guiding or trapping them. This occurs when a highly focused laser beam is incident on a transparent or semi-transparent dielectric object with a refractive index higher than that of the surrounding medium. The magnitude and direction of the induced force depend on the object’s optical and geometrical properties, namely its shape, size, refractive index and material composition.

Two primary components of optical force are identified: the scattering force, which acts along the beam’s propagation direction, and the gradient force, which pulls the particle toward the beam center where intensity is highest. Additional forces, such as radiometric and thermal forces, arise from interactions between the particle’s heated surface and its medium, leading to effects like thermophoresis and photophoresis. However, these forces are negligible at low pressure (Ashkin & Dziedzic, 1976 ▸; Hu *et al.*, 2023 ▸).

Today, this technique has evolved into a powerful tool known as optical tweezers, which utilize optical forces generated by continuous-wave (cw) and pulsed lasers to manipulate microscopic particles in colloidal physics and molecular biology (Chen *et al.*, 2022 ▸). Studies indicate that femtosecond pulsed lasers can be as effective as cw lasers, with average power, rather than peak power, being the key determinant of optical force (Xing *et al.*, 2004 ▸; Roy *et al.*, 2015 ▸; Preez-Wilkinson *et al.*, 2015 ▸). Femtosecond lasers offer distinct advantages when large instantaneous forces are required, such as for unsticking or deflecting particles (Agate *et al.*, 2004 ▸), as well as enabling nonlinear processes in particles (Goswami, 2021 ▸). Their nanoNewton-range gradient force further enhances capabilities for levitating and precisely controlling microscopic particles (Little *et al.*, 2004 ▸; Deng *et al.*, 2005 ▸; Ambardekar & Li, 2005 ▸; Jiang *et al.*, 2010 ▸).

Recently, vacuum-based optical tweezers have gained significant interest, particularly for trapping and cooling atoms (Li *et al.*, 2019 ▸; Gonzalez-Ballestero *et al.*, 2021 ▸; Spampinato *et al.*, 2024 ▸; Pichard *et al.*, 2024 ▸). Manipulating liquid droplets in vacuum is more challenging than manipulating solid particles due to evaporation. As droplets shrink, their radius *r* decreases, leading to an increase in Laplace pressure, Δ*p* = 2γ/*r*. This rise in internal pressure, driven by both the decreasing radius and ongoing evaporation, eventually destabilizes the droplet, complicating sustainable optical control (Malek *et al.*, 2018 ▸).

Though surface tension initially helps maintain droplet integrity for trapping, the rising internal pressure eventually destabilizes the droplet, complicating sustained optical manipulation. The following section explores these vacuum droplet dynamics in detail.

### 1.2. Liquid droplet dynamics in vacuum

Droplets can be generated through various methods, including short jet condensation into single droplets, droplet-on-demand mode, and continuous mode, where acoustic energy induces jet breakup (Minov *et al.*, 2015 ▸). The gas dynamic virtual nozzle technique utilizes coaxial gas flow to refine jet diameters, producing uniform 9–12 µm water droplets at hundreds of kHz via piezoelectric actuation (Sellberg *et al.*, 2014 ▸). The controlled breakup of cylindrical liquid jets, driven by surface free energy reduction, is crucial for producing uniform droplets with precise size, shape and velocity.

Injecting liquid droplet streams into vacuum environments has enabled a wide range of advanced studies, including Raman thermometry for analyzing the evaporative cooling of liquid water droplets (Smith *et al.*, 2006 ▸; Goy *et al.*, 2018 ▸; Takahashi *et al.*, 2024 ▸), investigations into microstructural evolution during the freezing of supercooled droplets (Kalita *et al.*, 2023 ▸), and ultrafast X-ray coherent scattering experiments on liquid-phase samples, such as pure water and solvent-diluted solutions (Sellberg *et al.*, 2014 ▸; Kim *et al.*, 2017 ▸; Yang *et al.*, 2021 ▸). Despite these advancements, such applications pose significant challenges due to the complex dynamics of liquid droplets, particularly their propagation and evaporation under vacuum conditions, where pressures are substantially lower than the liquid’s vapor pressure.

In vacuum, droplets undergo rapid evaporation due to the lack of external pressure, which removes heat and causes significant cooling, known as evaporative cooling. As energetic molecules escape, the liquid loses latent heat, further lowering its temperature. In extreme cases, rapid evaporation can lead to supercooling or freezing, a process widely applied in vacuum freeze-drying and observed in space environments.

For small droplets, evaporation is often described by the Hertz–Knudsen theory, which is based on kinetic evaporation principles and was experimentally validated by Knudsen (1915 ▸). Building on this, the framework of Smith *et al.* (2006 ▸) and Sellberg *et al.* (2014 ▸) extends the model originally introduced by Faubel *et al.* (1988 ▸), incorporating surface cooling effects. This approach treats the droplet as concentric spherical shells, where the outermost layer cools via evaporation while inner layers transfer heat through conduction, governed by the thermal gradient and the liquid’s thermal conductivity (for water, κ = 0.61 W m^−1^ K^−1^). When combined with molecular dynamics simulations, this model provides a robust framework for analyzing droplet evaporation and propagation in vacuum (Schlesinger *et al.*, 2016 ▸).

Expanding on these principles, a recent theoretical approach integrates mass and energy conservation to predict droplet size and temperature evolution during evaporative cooling (Guildenbecher *et al.*, 2021 ▸; Guildenbecher *et al.*, 2025 ▸). By estimating the mass and energy fluxes associated with evaporation and condensation, this method calculates molecular number change, energy loss and temperature variation in free-falling droplets. Solving these equations self-consistently enables precise predictions of droplet temperature and diameter throughout transit, offering critical insights into droplet dynamics under vacuum conditions. This approach has been adapted for the present study. While the model provides a solid foundation, it could be further refined. For instance, considering the droplet as a series of thin spherical shells, where the outer layers cool through evaporation and the inner layers transfer heat via conduction, governed by the thermal gradient, could offer a more detailed understanding and improve the accuracy of the model. However, such refinements are beyond the scope of the current study.

## 2. Theory and modeling

### 2.1. Impulse optical force modeling

When photons interact with an object, they transfer momentum, which can be calculated using Einstein’s relativistic equations,
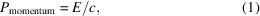
where *E* is the photon energy and *c* (= 3 × 10^8^ m s^−1^) is the speed of light in vacuum. In a medium with refractive index *n*_m_, the speed of light reduces to *c*/*n*_m_. When an object interacts with an incident light beam, the difference between the momentum flux entering and leaving the object induces a force, as described by Newton’s second law, while the energy rate of the beam corresponds to its power. Therefore, the optical force *F* [N] induced by a laser beam with power *P* [W] interacting with an object is given by
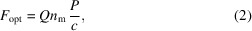
where *Q* (−1 < *Q* < 1) is a dimensionless factor influenced by the particle’s size, shape, refractive index and the incident beam properties such as the numerical aperture of the focusing lens, the laser power and the wavelength.

In contrast to a cw laser beam, which emits laser energy continuously over time, a pulsed laser concentrates the energy into short bursts. The pulse energy of the laser is given by
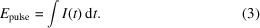
From equation (2), the instantaneous optical force from a laser pulse is
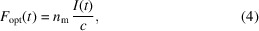
where *I*(*t*) is the pulse intensity, expressed as a function of time.

In other words, the force applied on a particle over a short time is called the impulse, related to the momentum change, Δ*P*, over time, *F*(*t*) = Δ*P*/Δ*t*, where Δ*t* is the pulse duration. Substituting this into equation (4) and accounting for the particle’s momentum (dependent on mass and velocity), we get 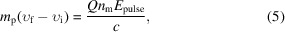
where *m*_p_ is the particle mass and (υ_f_ − υ_i_) represents the change in the particle’s velocity induced by the optical impulse.

Three main approaches are used for calculating the optical force depending on the particle size: geometrical optics for particles larger than 10λ, the Rayleigh approximation for those smaller than 0.1λ, and the intermediate regime for 0.1λ ≤ *d* ≤ 10λ (λ is the laser wavelength). Among these, the intermediate regime provides sufficient accuracy across all scales. The optical force induced on a particle due to momentum transfer is a light scattering problem, derived using linear momentum conservation from the scattered field distribution. The key step involves solving the scattering problem and calculating the Maxwell stress tensor to derive the optical force. The time-averaged optical force exerted by monochromatic light on a single particle is defined as
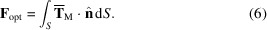
This integration is performed over a surface *S* enclosing the scatterer, where 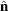 represents the outward unit normal vector. The term 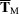 denotes the time-averaged Maxwell stress tensor, which comprehensively characterizes the electromagnetic interactions between light and matter. Further details on the Maxwell stress tensor and the calculation of optical forces can be found in Appendix *A*[App appa].

The generalized Lorenz–Mie theory (GLMT) and the transfer-matrix method are efficient for computing optical forces on spherical and non-spherical particles (Nieminen *et al.*, 2003 ▸; Waterman, 1971 ▸; Herranen *et al.*, 2019 ▸; Bui *et al.*, 2017 ▸; Gouesbet, 2019 ▸). The foundations of GLMT and the T-matrix methods are explained in Appendix *B*[App appb]. More details on the modeling approaches can be found elsewhere (Nieminen *et al.*, 2014 ▸; Pesce *et al.*, 2020 ▸; Burnham & McGloin, 2011 ▸; Bui *et al.*, 2017 ▸; Lenton *et al.*, 2018 ▸).

We used a MATLAB toolbox, developed by Nieminen *et al.*, for computational modeling of optical tweezers. This toolbox is designed to compute optical forces and torques for both spherical and non-spherical particles in Gaussian and other beam types. Additionally, it can be applied to light scattering calculations using Lorenz–Mie theory and the T-matrix method (Nieminen *et al.*, 2007 ▸; Lenton *et al.*, 2019 ▸).

### 2.2. Hertz–Knudsen theory for evaporation

The Hertz–Knudsen model provides equations for evaporation (ϕ_vap_) and condensation (ϕ_cond_) fluxes,
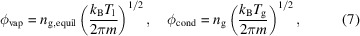
where *T*_l_ and *T*_g_ are liquid and gas temperatures, *k*_B_ is Boltzmann’s constant, and *m* is molecular mass. *n*_g,equil_ is the gas density in equilibrium, when *T*_l_ = *T*_g_, derived from vapor pressure *P*_vap_ and the ideal gas law. The local gas density *n*_g_ accounts for vapor from neighboring droplets and is expressed as
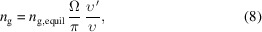
where Ω = 2π[1 − (1 − *d*^2^/4*a*^2^)^1/2^] represents the solid angle defined by the droplet diameter *d* and spacing, *a* = υ_*z*_/*f*, assuming υ_*z*_ as vertical velocity and a constant frequency *f* of the droplet streams. The υ′/υ term represents the fluid velocity ratio between the adjacent droplet surfaces and the droplet itself, approximating 1/2 for *a* ≫ *d*.

The evaporation rate, *J*_vap_, is defined as
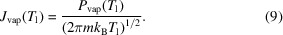
Mass conservation yields the droplet’s molecular count, *N*_l_, over time,
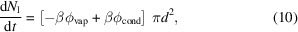
where β is the evaporation coefficient, representing the ratio of observed to theoretical maximum evaporation rates. It reflects the probability of a liquid molecule evaporating or a gas molecule condensing at the liquid–vapor interface. Deviations from β = 1 indicate kinetic or energetic barriers to molecular transfer. A theoretical expression relating β to molecular motion based on liquid and vapor densities was derived by Nagayama & Tsuruta (2003 ▸) as 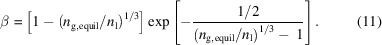
The temperature dependence of β shows minimal variation (10% or less), having little effect on the temperature and diameter changes of the droplets. Recent studies suggest β = 1 for water droplets (Persad & Ward, 2016 ▸), with molecular dynamics simulations confirming this under various conditions (Sellberg *et al.*, 2014 ▸).

Using equations (7) and (8) to rewrite (10) and then substituting (9) yields the rate of molecule numbers change in a droplet,
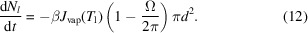
The droplet diameter is derived from the total molecule count, as
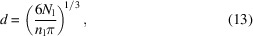
where *n*_l_ = ρ_l_/*m* is the liquid number density, with ρ_l_(*T*_l_) being the liquid density. Conservation of energy, assuming thermal equilibrium *T*_l_ = *T*_g_, can be expressed as
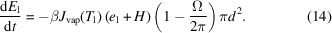
In this equation, *E*_l_ is the total energy of the droplet, and *e*_l_ is the average thermal energy per molecule lost to the gas at liquid temperature *T*_l_, given by 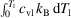. Here, *c*_vl_ is the specific heat of the liquid, which is used in a dimensionless form in this approach, and *H* represents the enthalpy of vaporization. The average energy per liquid molecule, ɛ_l_ =*E*_l_/*N*_l_, is related to the liquid’s specific heat by dɛ_l_/d*T*_l_ = *k*_B_*c*_vl_ which results in the droplet temperature *T*_l_ evolution as 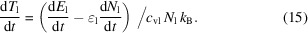
Coupled ordinary differential equations for *N*_l_, *E*_l_ and *T*_l_, equations (12)–(15), govern the change of droplet diameter and temperature during vacuum travel. Solving these equations requires initial values of droplet diameter, velocity and temperature.

#### 2.2.1. Study case: water

**Figure 1:**
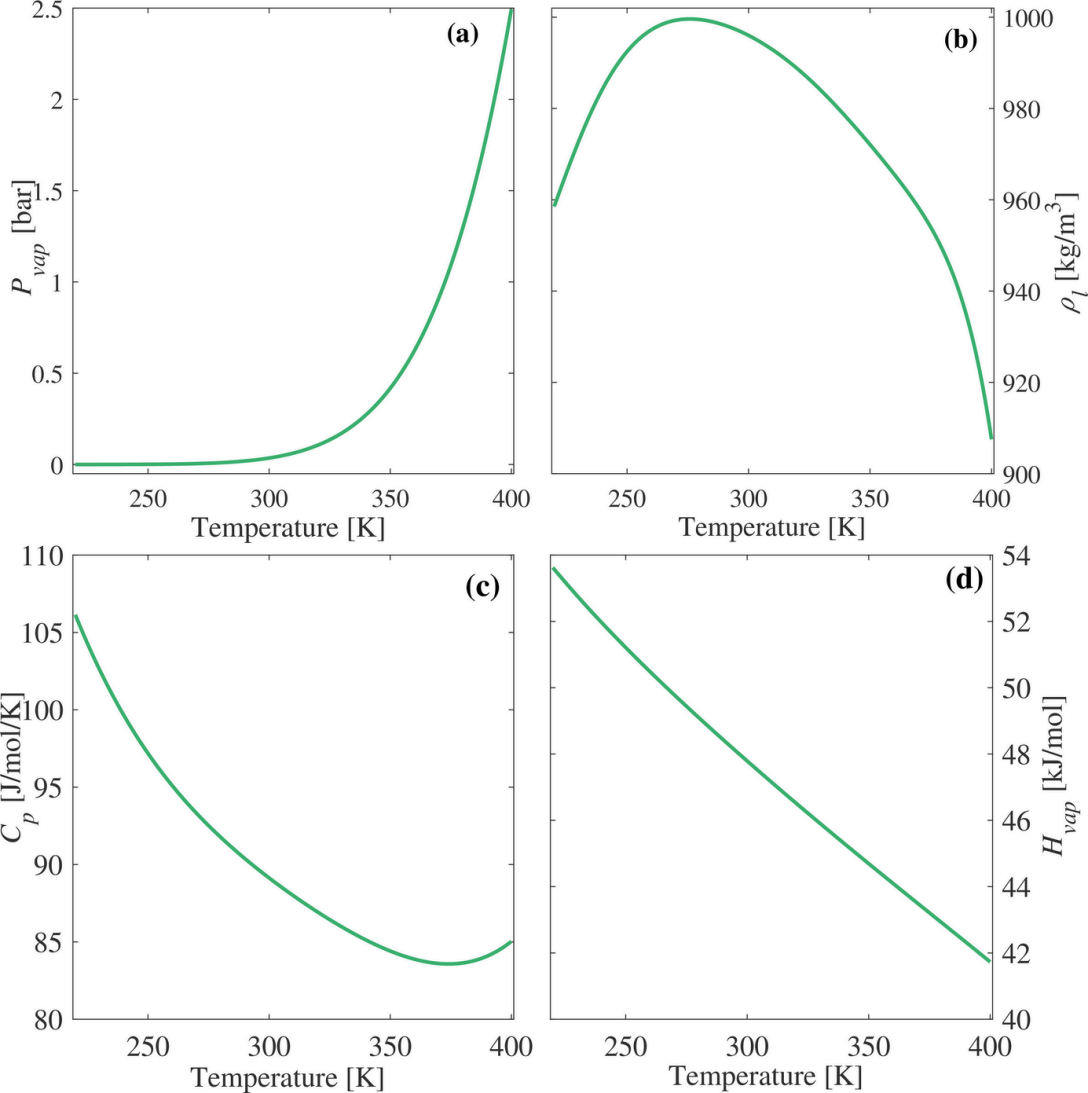
Temperature evolution of the thermodynamics properties of the TIP4P/2005 water model: (*a*) vapor pressure, (*b*) density, (*c*) specific heat capacity and (*d*) enthalpy of vaporization.

The temperature variation of thermodynamic properties for the TIP4P/2005 water model, with a molar mass of 18 g mol^−1^, is presented in Fig. 1 ▸. Molecular dynamics simulations, fitted with polynomial functions (Schlesinger *et al.*, 2016 ▸), have been applied to determine the temperature dependence. The analyzed properties include density, specific heat capacity and enthalpy of vaporization. The Murphy and Koop equation (Kalova & Mares, 2010 ▸) has been used to describe water vapor pressure changes with temperature.

The pressure inside small droplets increases due to the high curvature of the droplet surface and the effects of surface tension. Consequently, the vapor pressure also rises and can be modified using the Kelvin equation, *p*_vap_ = 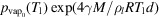, where 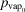 represents the vapor pressure given by the Murphy and Koop equation for a flat interface, the surface tension of water is γ = 0.0756 N m, at *T*_l_ = 273 K, *M* is the molar mass and *R* = 8.31 J mole^−1^ K^−1^ is the universal gas constant. The reported molecular dynamics simulations confirm that the exponential correction factor is significant only at sub-micrometre scales, particularly below 10 nm (Schlesinger *et al.*, 2016 ▸), indicating that the correction to *p*_vap_ is negligible for micrometre-sized droplets. Moreover, experiments suggest that evaporation-induced pressure and density gradients can cause horizontal spreading in a free-falling droplet stream, an effect amplified at higher nozzle temperatures (Guildenbecher *et al.*, 2021 ▸). However, this effect diminishes with larger droplet spacing (Crowe, 2005 ▸; Guildenbecher *et al.*, 2021 ▸).

**Figure 2:**
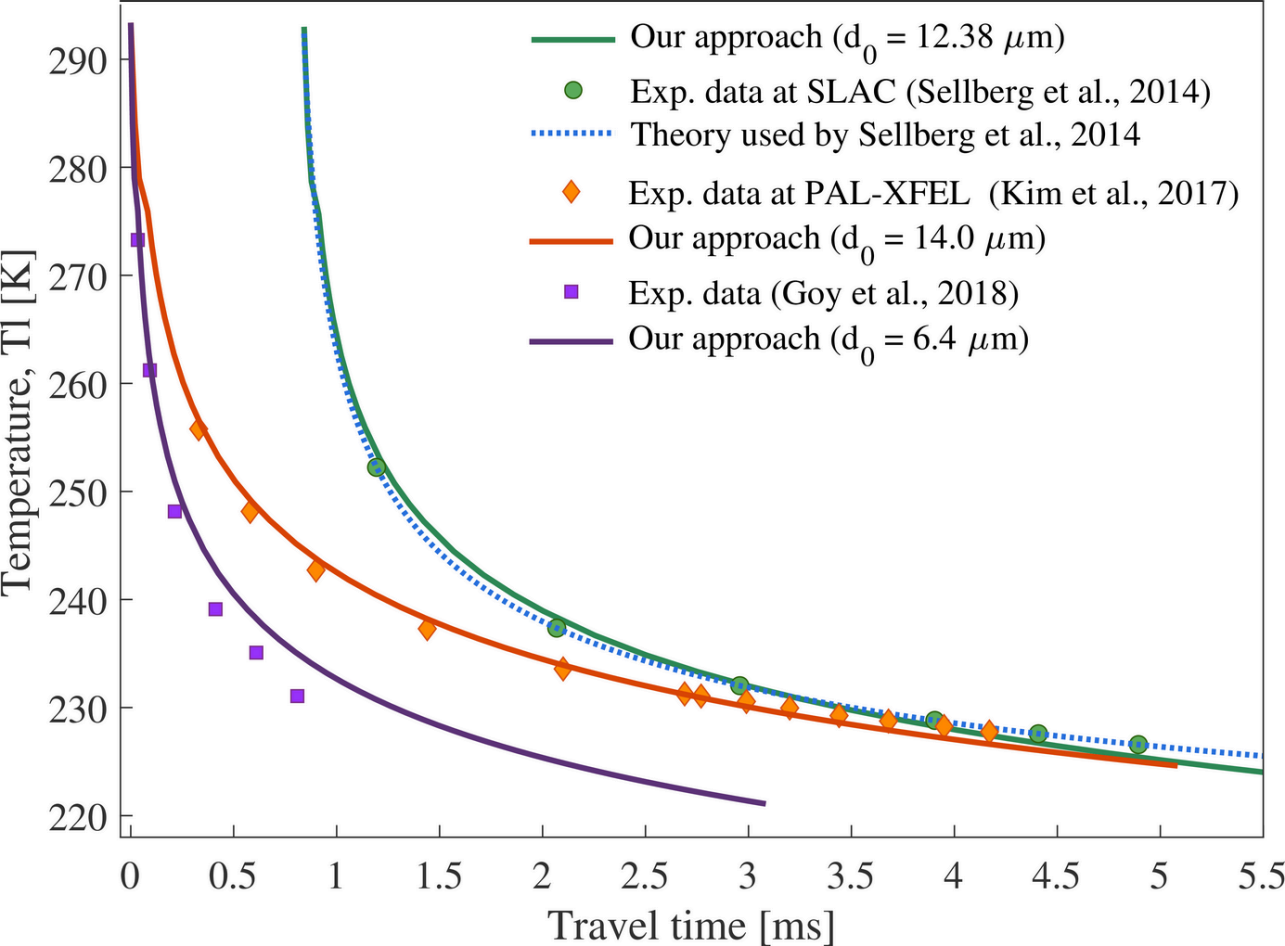
Temperature change of free-falling water droplets during evaporative cooling in vacuum; comparison of predicted values (solid lines) with theoretical prediction (blue dotted line) and experimental data for droplets with *d*_0_ = 12.38 µm, ν_*z*0_ = 10.35 m s^−1^ (green circles), *d*_0_ = 14.0 µm, ν_*z*0_ = 15.3 m s^−1^ (orange diamonds) and *d*_0_ = 6.4 µm, ν_*z*0_ = 22 m s^−1^ (purple squares)

Fig. 2 ▸ presents the predicted temperature values from the adapted Hertz–Knudsen model (solid lines) (Guildenbecher *et al.*, 2021 ▸), compared with the theoretical approach from Smith *et al.* (2006 ▸) and Sellberg *et al.* (2014 ▸) (blue dotted line), which introduces a thermal gradient from the bulk to the droplet’s surface. The comparison shows good agreement, validating our adapted model and suggesting that, due to the small droplet size, the formation of a significant temperature gradient is minimized.

Additionally, in Fig. 2 ▸, the predicted temperature evolution is compared with experimental data from SLAC (Sellberg *et al.*, 2014 ▸) and PAL-XFEL (Kim *et al.*, 2017 ▸) for droplets with initial sizes and velocities of *d*_0_ = 12.38 µm, ν_*z*0_ = 10.35 m s^−1^ (green circles) and *d*_0_ = 14.0 µm, ν_*z*0_ = 15.3 m s^−1^ (orange diamonds), showing very good agreement. Further comparison was made with experimental temperature data for droplets of *d*_0_ = 6.4 µm, ν_*z*0_ = 22 m s^−1^, obtained via Raman spectroscopy, where the shape of the Raman O—H stretching bands was correlated with droplet temperature (Goy *et al.*, 2018 ▸). Deviations from the theoretical model were attributed to increased scattering, possibly caused by droplets that froze into ice during evaporative cooling in the vacuum. The initial temperature for all experiments and simulations was defined as *T*_l_ = 293 K.

## 3. Results and discussion

**Figure 3:**
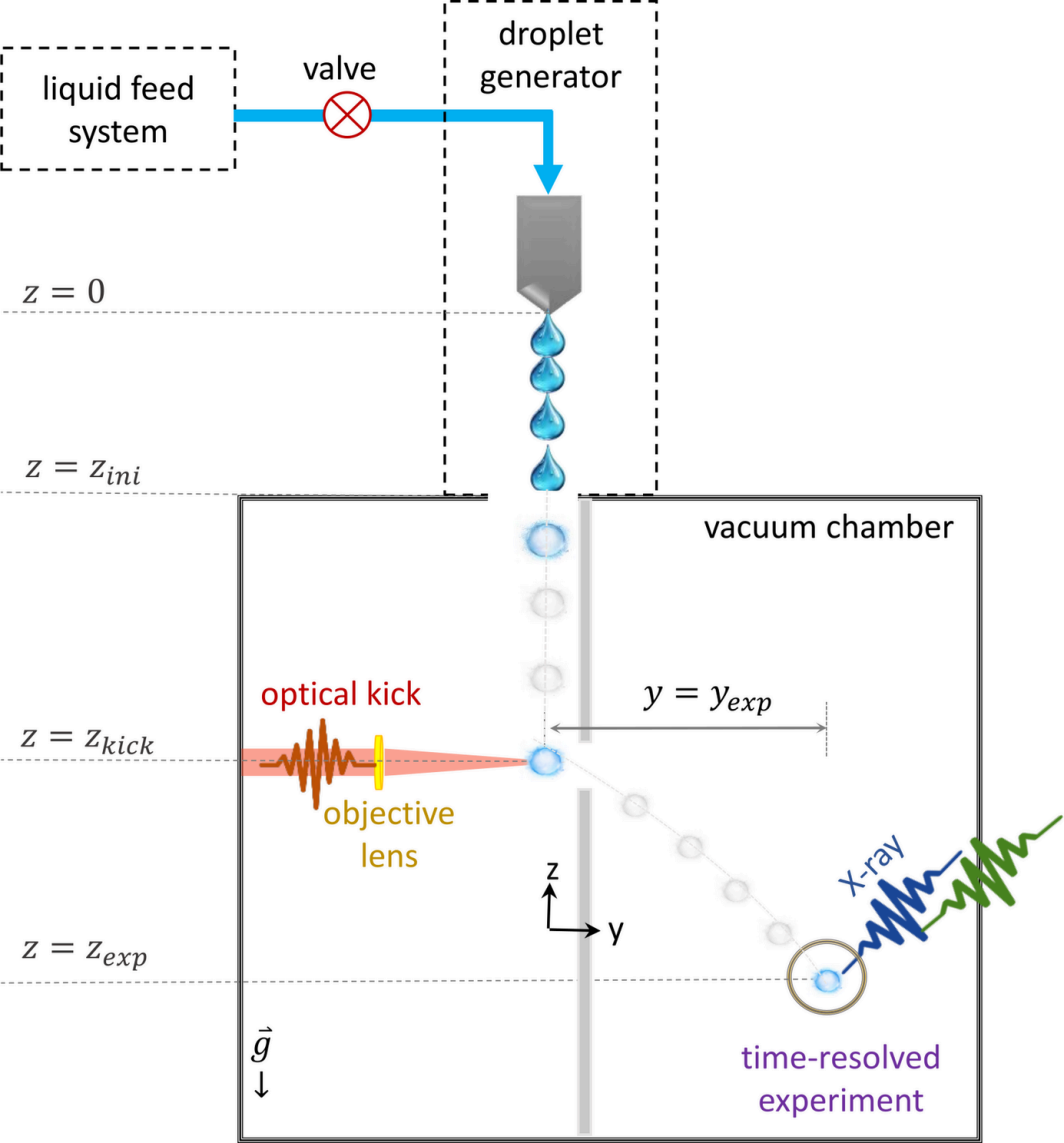
Schematic of the liquid sample delivery setup. A droplet generator produces a train of single droplets falling under gravity into a vacuum chamber. At *z* = *z*_kick_, a femtosecond pulse laser induces transverse momentum (an optical kick) to direct a single droplet toward the time-resolved X-ray experiment point at *z* = *z*_exp_, *y* = *y*_exp_. The gray lines and droplets represent the trajectory toward the interaction region.

Building upon the discussions in the preceding sections, we propose a conceptual design for a compact liquid sample delivery system, tailored for use in soft X-ray experiments. This design is particularly suited for ultrafast free-electron laser beamlines, offering efficient delivery and alignment of liquid samples under vacuum conditions. A schematic layout of the proposed system is illustrated in Fig. 3 ▸. The setup includes (i) a portable vacuum chamber equipped with optical windows to allow laser passages, ensuring compatibility with high-vacuum experimental requirements, (ii) a droplet generation system in order to produce a liquid droplets stream with precise control over size and spacing, (iii) a femtosecond laser in order to provide the optical force. The beam is focused through an objective lens with high numerical aperture. Finally, (iv) the experimental interaction zone where the soft X-ray beam intersects with the liquid droplets.

A liquid droplet train (*e.g.* water) is generated in this design with a defined frequency and initial velocity, υ_*z*0_. The droplets are introduced into the high-vacuum chamber at *z* = *z*_ini_, with an initial size of *d*_0_ and temperature *T*_l0_. During the transit in the vacuum, their size and temperature decrease due to evaporation phenomena. The free-falling micro-droplet, now with a size of *d*_1_ and temperature *T*_l1_, experiences an optical kick perpendicular to its propagation direction. This kick is induced by a focused pulse laser with defined parameters, delivering a force with a strength depending on the particle size and location upon reaching the laser at *z* = *z*_kick_. As a result of this impulse force the micro-droplet acquires a velocity υ_*y*_ in the direction of the force.

Following this new trajectory, the droplet is directed to the interaction point of the ultra-fast soft X-ray experiment, arriving at (*z*, *y*) = 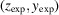 with a predicted size and temperature. This enables improved experimental accuracy and more reliable sample delivery.

### 3.1. Simulation algorithm

This section outlines the computational procedure developed to simulate the trajectory and thermodynamic evolution of a liquid droplet from generation to interaction with the ultra-fast soft X-ray beam. The algorithm incorporates liquid droplet dynamics, heat and mass transfer, and optical force calculations, providing a step-by-step framework to estimate the droplet’s state at critical points along its path.

**Step 1***Definition of parameters for droplet generation.* The frequency *f* and initial velocity ν_*z*0_ determine the inter-droplet spacing, given by *a* = ν_*z*0_/*f*. Initial properties, including the size *d*_0_ and temperature *T*_l0_, need to be specified.

**Step 2***Estimation of droplet size at laser interaction.* The size *d*_1_ and temperature *T*_l1_ at time *t*_1_ and position *z* = *z*_kick_ which is the nozzle–laser distance are quantified by solving the Hertz–Knudsen equations, as detailed in Section 2.2, using the fourth/fifth-order Runge–Kutta (RK45) method. This requires knowing the temperature-dependent thermodynamic properties of the liquid, including the density, specific heat capacity, vapor pressure and enthalpy of vaporization.

**Step 3***Calculation of the laser-induced optical force efficiency, Q.* Given the droplet size *d*_1_ and the refractive index, *Q* is calculated using the Lorenz–Mie theory and the transfer matrix method, introduced in Section 2.1 and Appendices *A*[App appa] and *B*[App appb], as a function of the distance from the laser’s focal plane. The impulse optical force applies a constant velocity ν_*y*_ to the droplet, as described in equation (5). At this stage, the droplet travels along the *y*-axis while continuing to fall due to gravity. The simple position–velocity equation is applied to estimate the time *t*_2_ = *y*_exp_/ν_*y*_, which is the time required for the droplet to reach the experimental interaction point.

**Step 4***Estimation of final state of droplets.* The Hertz–Knudsen equations are solved again at time *t*_2_ to estimate the final droplet diameter *d*_2_ and temperature *T*_l2_ at the interaction point. When solving these equations, the updated initial conditions—temperature *T*_l1_, diameter *d*_1_ and velocity υ = 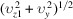
—are used. The droplet frequency matches the repetition rate of the kicking laser, *e.g.f* = 50 Hz. In the case of liquid phase experiments, we need to ensure that the total travel time satisfies the supercooled liquid stability condition, *t*_1_ + *t*_2_ < *t*_liquidphase_, which represents the maximum allowable time to maintain the droplet in its liquid phase during the experiment.

### 3.2. Water droplet manipulation in vacuum

We assume that a periodic stream of uniform ultrapure liquid water micro-droplets are injected into the proposed delivery system with defined initial nozzle temperature and droplet size. Droplet size and velocity are determined from the volume flow rate and droplet parameters. The flow rate is related to the jet diameter, speed and frequency. Additionally, the droplet speed can be calculated from the spacing between droplets and the frequency of their formation (Sellberg *et al.*, 2014 ▸). In our example case, water droplets are generated by applying external excitation at a frequency of *f* = 186 kHz with an initial velocity of υ_*z*0_ = 15.3 m s^−1^. This is achieved using a piezoelectric actuator to induce jet breakup. The initial conditions are adapted from experimental data reported by Kim *et al.* (2017 ▸).

**Figure 4:**
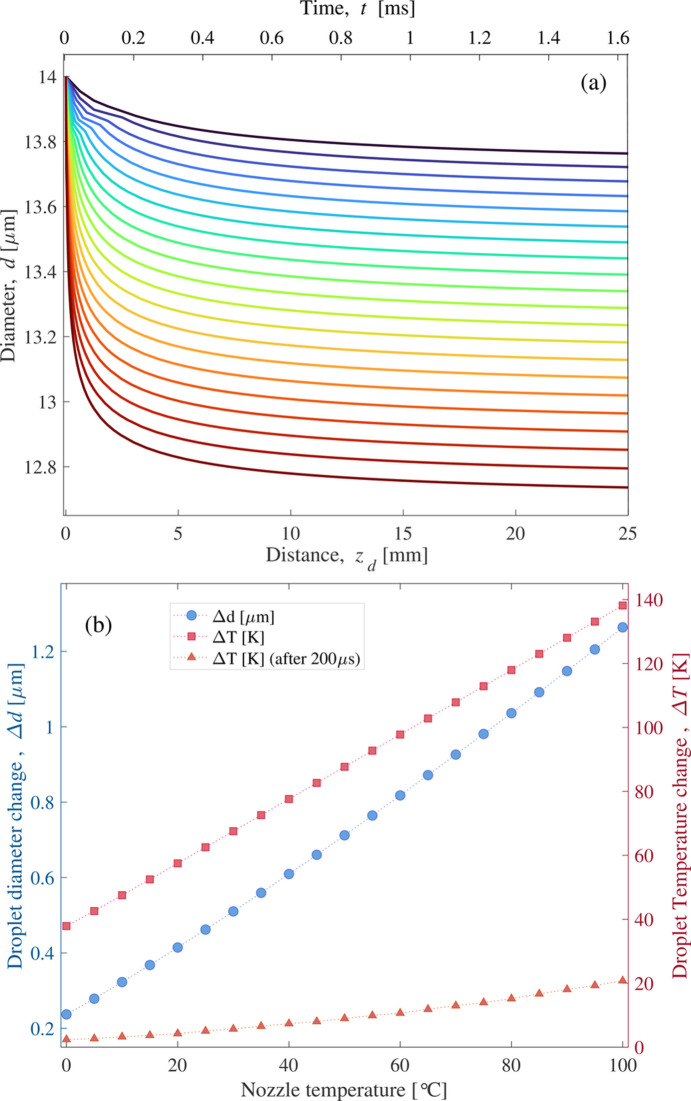
(*a*) Predicted evolution of droplet diameter for liquid water droplets with *d*_0_ = 14 µm, υ_*z*0_ = 15.3 m s^−1^, *f* = 186 kHz and nozzle temperatures ranging from 0°C to 100°C (corresponding to the line colors). (*b*) Droplet diameter (blue circles) and temperature changes (red squares) as a function of nozzle temperature. Red triangles represent evaporative cooling of the water droplet after 0.2 ms of travel in a vacuum.

The impact of the initial temperature on droplet size change and cooling in a vacuum is illustrated in Fig. 4 ▸, for droplets with an initial diameter of *d*_0_ = 14 µm. Lower temperatures reduce size loss due to evaporative cooling. Simulations confirm that initial temperature has little impact on final supercooled water temperature, as most cooling happens within ∼200 µs, with higher nozzle temperatures speeding up the process. Moreover, an experiment by Guldenbecher *et al.* (2021 ▸) showed that higher nozzle temperatures increase the horizontal spread of droplets in falling stream experiments. This is due to gas-phase forces from evaporation-induced pressure gradients, which push droplets outward. Higher temperatures amplify this effect by increasing vapor pressure. To minimize these forces, keeping the nozzle temperature as low as possible is recommended.

**Figure 5:**
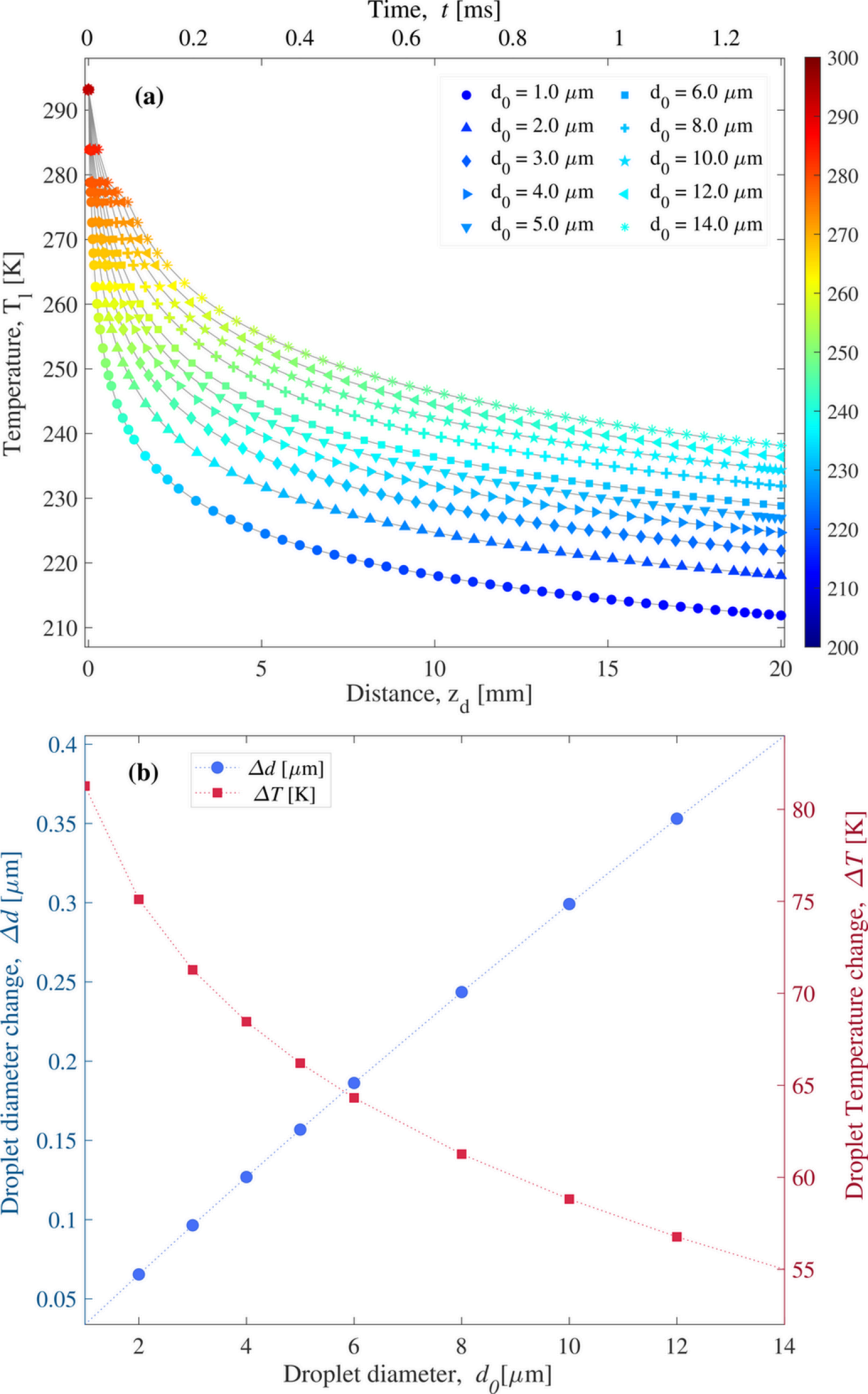
(*a*) Evaporative cooling behavior and (*b*) diameter and temperature change of free-falling water droplets with varying initial diameters, starting at *T*_l0_ = 293 K and υ_*z*0_ = 15.3 m s^−1^.

Fig. 5 ▸ shows the temperature change of free-falling droplets in a vacuum for sizes ranging from 1 µm to 14 µm, while keeping the initial temperature, velocity and droplet stream frequency constant at *T*_l0_ = 293 K, υ_*z*0_ = 15.3 m s^−1^ and *f* = 186 kHz, respectively. The total change of diameter and temperature change of a free-falling water droplet after traveling a distance of *z* = 20 mm is presented in Fig. 5 ▸(*b*). The results demonstrate that larger droplets exhibit more mass loss and less temperature change due to evaporative cooling.

The optical kicker is positioned below the nozzle in vacuum, at 20 mm in our simulation, considering experimental alignment and setup constraints on the order of millimetres. Since the refractive index of water remains nearly constant across optical wavelengths, flexibility is maintained. The femtosecond pulse laser with wavelength and pulse duration of λ = 780 nm and τ = 100 fs, respectively, propagates along the *y*-axis. The beam is focused by an objective lens with a numerical aperture NA of 0.5, which sets the beam focal size, *w*_0_ ≃ 0.61λ/NA. A short focal length should be avoided to reduce setup complexity. If the laser spot is smaller than the particle, scattering forces dominate, causing an optical kick (Ashkin, 1980 ▸; Jonáš & Zemánek, 2008 ▸). When this condition is met, the pulse-transferred-momentum induces velocity in the impulse direction.

**Figure 6:**
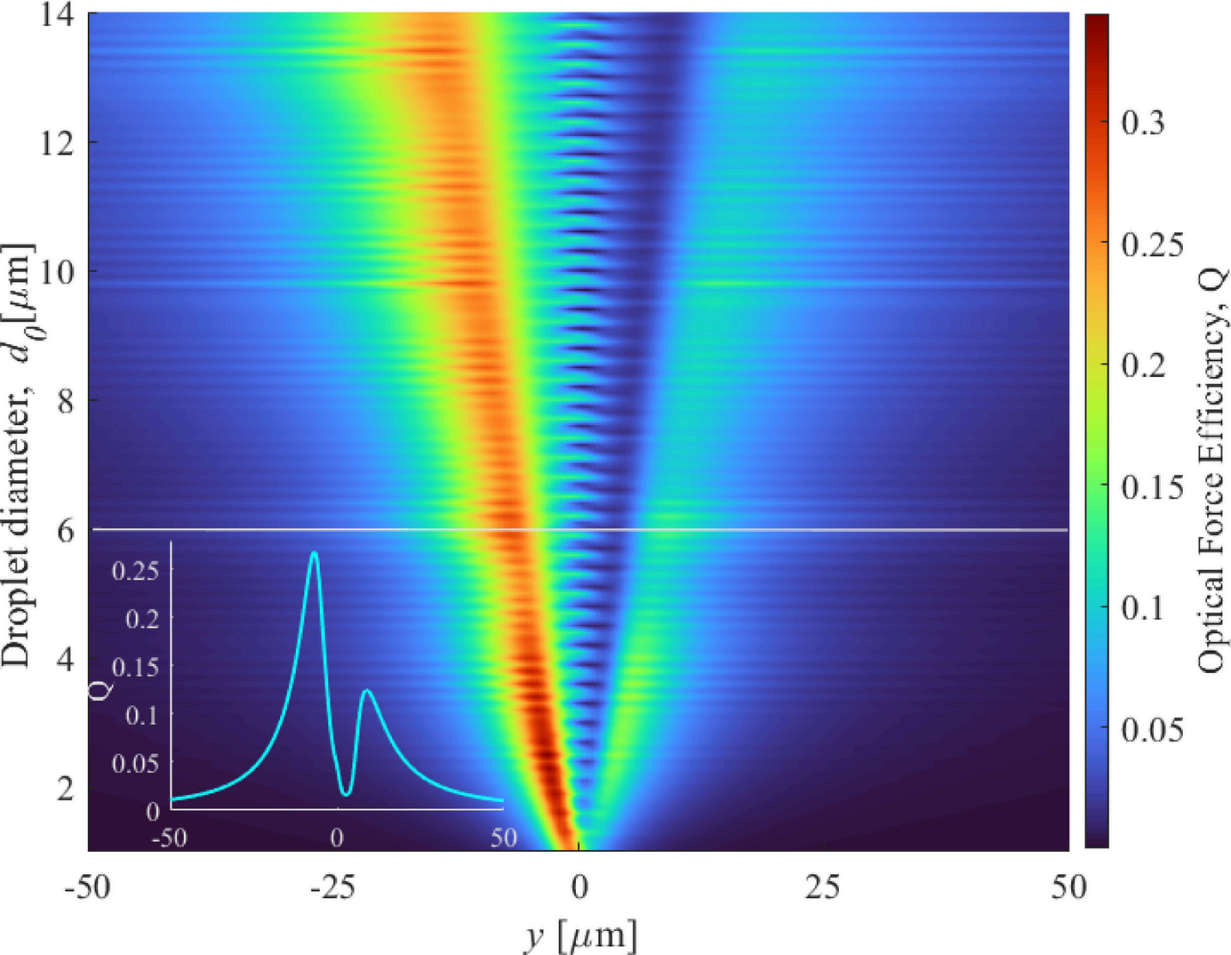
The optical efficiency *Q* as a function of the distance *y* from the focal point of a circularly polarized Gaussian femtosecond laser (λ = 780 nm, NA = 0.5); the maximum force efficiency occurs just before the focal plane. Interaction occurs with evaporating water droplets (*n*_water_ = 1.3232) in a vacuum, with varying droplet sizes. The inset displays the *Q* profile for a particle size of *d*_0_ = 6 µm.

The optical force efficiency, *Q*, as a function of the distance from the focal plane, is presented in Fig. 6 ▸, for different initial droplet diameters. The inset of the figure shows the *Q*-profile for a droplet with an initial diameter of *d*_0_ = 6 µm. Notably, the induced force does not change sign in the region around the focus, indicating the absence of axial equilibrium and, consequently, the lack of stable trapping within the studied range. Instead, the laser pulse applies a unidirectional force, pushing the droplets along the beam propagation direction. The maximum force efficiency occurs before the focal plane, which is represented as *y* = 0.

Interestingly, as the initial droplet size increases, the position of the force maximum shifts toward negative *y*-values, while the force efficiency decreases correspondingly. For instance, droplets with a diameter of *d*_0_ = 6 µm exhibit a maximum force efficiency of 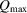 = 0.27, occurring at *y* = −7 µm, inducing a velocity of υ_*y*_ = 8.95 m s^−1^ to the particle. Furthermore, for smaller droplets, the *Q*-values display a significant peak at positive *y*. Given these values and the pulse energy, the motion of the droplets in a vacuum can be precisely controlled and directed toward the experimental interaction point.

The positioning of the droplets relative to the focus point is determined by the force required to deliver the sample to the experimental interaction region. Using equation (5), the induced velocity of the droplets can be calculated. In our simulation, we utilize the maximum *Q* value and its corresponding position. During femtosecond laser-droplet interaction, thermal diffusion is negligible due to the short pulse duration, which is significantly smaller than the heat propagation timescale (ps) for water.

**Figure 7:**
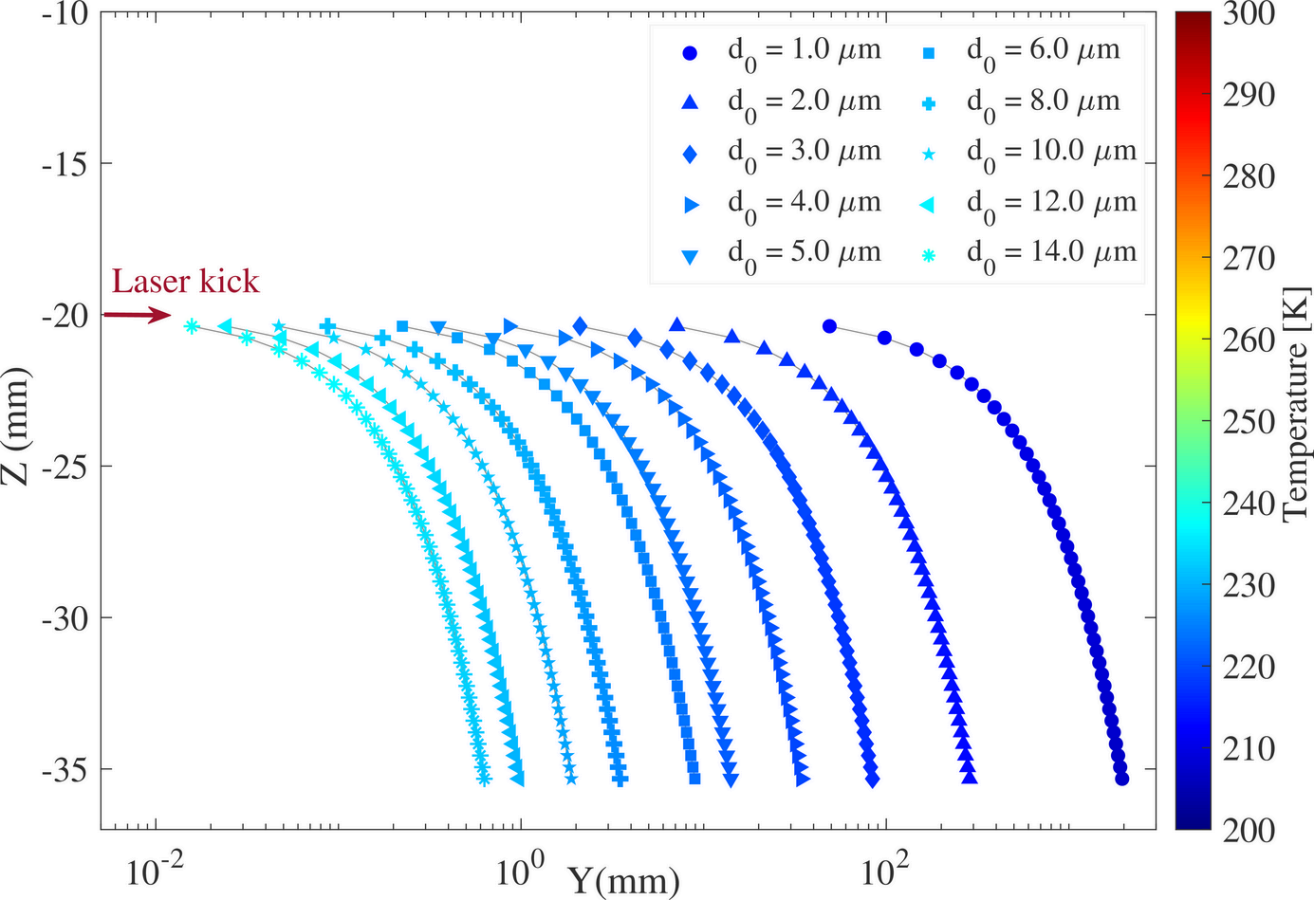
Traveling path of a falling water droplet with different sizes after receiving a kick from a 50 Hz femtosecond laser, *E*_pulse_ = 1 mJ, λ = 780 nm, focused with an objective of NA = 0.5. The travel time after the kick has been fixed to be *t* = 1 ms.

The travel path of droplets of varying sizes after receiving the optical kick, along with their corresponding temperature changes, is presented in Fig. 7 ▸. The travel time after the laser kick is fixed at 1 ms. The figure demonstrates that the momentum-induced impulse force applied by the kicking laser is greater for smaller droplets, causing them to travel farther, while their fall in the *z*-direction remains similar.

As shown in Fig. 4 ▸, both droplet size and temperature remain relatively unchanged after the first 500 µs of travel in a vacuum. However, Fig. 7 ▸ reveals that initially larger droplets reach the experimental stage at a higher temperature. Given the fixed position of the sample in the ultra-fast experiment, proper adjustments to the laser power and focal point relative to the falling droplet can be made to modify the impulse values and, consequently, the induced velocity. This allows for effective handle of droplets in different sizes.

It is important to note that, when conducting experiments on liquid-phase droplets in a vacuum, the total travel time of a water droplet must be limited to a few milliseconds. Studies have shown that the freezing process can begin in approximately 3 ms and be completed in less than 5 ms (Sellberg *et al.*, 2014 ▸). However, these values may vary depending on experimental conditions such as vacuum parameters, droplet generation methods and other influencing factors. For instance, Goy *et al.* (2018 ▸) observed droplet freezing occurring as early as 1.2 ms.

The optical kicker, operating at a low repetition rate (50 Hz in our case), effectively isolates the droplet from others, preventing the formation of vapor pressure gradients around it. To ensure precise sample delivery, the laser must be properly synchronized with the experimental lasers so that the droplet reaches the interaction position exactly at the time of the laser pulses.

## 4. Conclusions and outlook

In this work, we proposed an optical force based technique for manipulating liquid droplets in vacuum, offering a reliable method for ultra-fast soft X-ray experiments. We developed a liquid sample delivery method using a femtosecond pulsed laser to precisely direct droplets to the experimental inter­action point. Simulations based on the Hertz–Knudsen equations and Lorenz–Mie theory estimated droplet thermodynamic behavior and required optical force, which can be adjusted by tuning laser parameters.

This approach provides a robust platform for studying supercooled micrometre-sized liquid droplets and phase transition processes at extremely low temperatures. The key advantage of optical kicking for sample delivery is the minimized sample waste, as it reduces the consumption of expensive or rare biological specimens compared with traditional liquid jets. Additionally, it ensures precise control by allowing droplets to interact with X-rays at the optimal position, enhancing data quality. Another advantage is flexibility in sample delivery, as adaptive droplet positioning reduces clogging and contamination risks, unlike static nozzles. The system is compact and adjustable for various ultrafast soft X-ray experiments using conventional lasers or large-scale facilities like free-electron laser and high harmonic generation sources, though further refinements are needed for optimal performance.

This method can be extended to other liquids, as long as their thermodynamic properties and refractive indices are characterized. Future enhancements include integrating a user interface for improved usability and incorporating machine learning for predictive modeling of evaporative cooling and optical kick effects. These advancements will enhance the system’s capabilities for ultrafast science and precision-controlled sample delivery in high-vacuum environments.

## Data Availability

The data that support the findings of this study are available from the corresponding author upon reasonable requests

## Appendix A Optical force calculation

The time-averaged optical force exerted by monochromatic light on a single particle is defined as

The integration is over a surface *S* around the scatterer, with  as the outward unit normal.  is the time-averaged Maxwell stress tensor, describing light–matter electromagnetic interactions. For harmonic fields, the Maxwell stress tensor is given by the total electric and magnetic field amplitudes **E**_t_ (= **E**_i_ + **E**_s_) and **B**_t_ (= **B**_i_ + **B**_s_), representing the sum of incident (**E**_i_, **B**_i_) and scattered (**E**_s_, **B**_s_) fields,

where ⊗ denotes the dyadic (outer) product and **I** is the unit outer product. In the far-field region, the expressions for the radiation force can be significantly simplified. By integrating over a spherical surface of large radius (*r* → ∞), fast-decaying terms can be neglected, leading to

where the integration is over the full solid angle (Ω = 4π). These expressions form the basis for electromagnetic calculations of optical forces, particularly in the intermediate size regime. The key step is solving the scattering problem by computing the scattered fields and the corresponding Maxwell stress tensor, from which the optical force is derived, as shown in equations (16) and (18).

## Appendix B Transfer matrix method

We can write the incident and scattered fields in terms of a discrete basis set of functions  and  where *n* and *k* are the mode index labeling the functions, respectively, each of which is a solution of the Helmholtz equation,

where *a*_*n*_ and *s*_*k*_ are the expansion coefficients for the incident and scattered waves, respectively. As long as the response of the scatterer, *i.e.* the particle, is linear, the incident and scattered fields must be linearly related, and the field coefficients can be expressed using a simple matrix equation,

where *T*_*kn*_ are the elements of the transfer matrix *T* that relates the incident and scattered field coefficient vectors

For spherical particles, the transfer matrix is diagonal, with its entries being the Mie coefficients, which can be calculated rapidly.
